# A Novel Approach of Antiviral Drugs Targeting Viral Genomes

**DOI:** 10.3390/microorganisms10081552

**Published:** 2022-07-31

**Authors:** Phuong Thi Hoang, Quynh Xuan Thi Luong, Ramadhani Qurrota Ayun, Yongjun Lee, Thuy Thi Bich Vo, Taehyun Kim, Sukchan Lee

**Affiliations:** 1 Department of Integrative Biotechnology, Sungkyunkwan University, Suwon 16419, Gyeonggi-do, Korea; hoangphuong06cs@gmail.com (P.T.H.); quynh.ltx2017@gmail.com (Q.X.T.L.); ramadhani.qurrota@gmail.com (R.Q.A.); 88yjl11@naver.com (Y.L.); bichthuy251188@gmail.com (T.T.B.V.); 2 Novelgen Co., Ltd., R&D Center, 77, Changnyong-daero 256 Beon-gil, Yeongtong-gu, Suwon 16229, Gyeonggi-do, Korea; thkim@novelgen.co.kr

**Keywords:** viral disease, viral genome degradation, broad-spectrum antiviral drugs, 3D8 scFv, ISGs, CRISPR/Cas, antiviral antibodies, RNAi

## Abstract

Outbreaks of viral diseases, which cause morbidity and mortality in animals and humans, are increasing annually worldwide. Vaccines, antiviral drugs, and antibody therapeutics are the most effective tools for combating viral infection. The ongoing coronavirus disease 2019 pandemic, in particular, raises an urgent need for the development of rapid and broad-spectrum therapeutics. Current antiviral drugs and antiviral antibodies, which are mostly specific at protein levels, have encountered difficulties because the rapid evolution of mutant viral strains resulted in drug resistance. Therefore, degrading viral genomes is considered a novel approach for developing antiviral drugs. The current article highlights all potent candidates that exhibit antiviral activity by digesting viral genomes such as RNases, RNA interference, interferon-stimulated genes 20, and CRISPR/Cas systems. Besides that, we introduce a potential single-chain variable fragment (scFv) that presents antiviral activity against various DNA and RNA viruses due to its unique nucleic acid hydrolyzing characteristic, promoting it as a promising candidate for broad-spectrum antiviral therapeutics.

## 1. Introduction

Viral infections pose a major risk to global health. The most recent case is the coronavirus disease 2019 (COVID-19) pandemic caused by the severe acute respiratory syndrome coronavirus 2 (SARS-CoV-2), which has infected more than 513 million people worldwide, with 6.2 million deaths as of May 2022 [1]. Other coronavirus strains cause life-threatening diseases such as SARS and the Middle East respiratory syndrome (MERS) [2,3]. Re-emerging virus infection, such as the Ebola virus, was reported in West Africa in 2014–2016, causing an outbreak with a case fatality rate of up to 50%, and the most recent case, which is still ongoing, has been reported in the Democratic Republic of Congo with a case fatality rate of 66% [4]. In April 2022, new Ebola virus cases were reported in the Mbandaka, Equateur Province of the Democratic Republic of Congo, indicating that the Ebola virus remains a major threat to global health [5]. On the other hand, viral infections caused by human immunodeficiency virus (HIV), hepatitis B virus (HBV), and hepatitis C virus (HCV) continue to pose a threat to global health. Globally, it is reported that approximately 1.7 million people have been newly infected with HIV, with 700,000 HIV-related deaths [6]. In addition, approximately 250 million and 71 million people have been reported to have chronic HBV and HCV, respectively, and the death toll caused by this viral hepatitis is estimated at approximately 1.34 million [7]. Flu caused by the influenza virus is also a global health issue yearly. In 2009, a global pandemic caused by the influenza A (H1N1) pdm09 virus resulted in more than 150,000–570,000 deaths worldwide, and it is currently circulating as a virus causing seasonal flu [8]. Avian influenza A (H5N1), another variant of influenza viruses, has become a concern since its first human infection in 1997 [9]. The viral epidemic and pandemic have caused many casualties throughout history, which are summarized in Table 1.

The viral life cycle consists of several major stages, including virus attachment to the membrane of the host cell, viral entry (i.e., endocytosis), viral uncoating, viral genome transcription and replication, viral proteins translation, viral assembly, and viral budding/release. The mechanisms of viral transcription and replication vary depending on their genome type (RNA/DNA; single- or double-stranded; and positive (+) or negative (−) polarity) [10]. Notably, regardless of the virus type, all viruses must release the viral genomes to the cytoplasm, produce mRNA, and replicate their genomes. Most DNA viruses transcribe their genome in the nucleus of the host cells and use the host enzymes for their DNA replication and gene expression; except the Poxvirus such as the Vaccinia virus, which can independently replicate in the cytoplasm because of its viral genome that encodes viral proteins responsible for transcription and replication processes [11]. The RNA viruses replicate their genome in the cytoplasm of the host cell with the help of RNA-dependent-RNA polymerase (RdRp). However, in retroviruses such as HIV, the RNA genome is reversely transcribed by RNA-dependent-DNA polymerase (RdDp) and subsequently delivered to the nucleus to integrate with the host genome [12,13]. Another example of RNA viruses replicating within the nucleus is influenza viruses [14].

The stages of the viral life cycle have become a common target for the development of antiviral drugs against viral or host cell proteins to inhibit viral replication [10,15]. Although numerous approaches against viral infection have been developed and approved, the fact that drug-resistant virus strains are increasing has made it difficult to use current drugs for treating viral diseases [16,17,18,19,20]. Therefore, in an effort to discover new drugs capable of combating a broad spectrum of viruses, we introduce a novel platform that directly targets viral genome degradation to inhibit viral replication. Here, to the best of our knowledge, we reviewed all candidates that show antiviral effects in the in vitro and in vivo tests of viral genome cleavage, including ribonucleases (RNases), interferon (IFN)-stimulated genes 20 (ISG20), RNA interference (RNAi), CRISPR/Cas systems, and a catalytic single-chain variable fragment 3D8 (3D8 scFv). They presented as the most straightforward and universal strategies for cleaving the viral genomes.

## 2. Antiviral Approach Targeting Viral Genomes

### 2.1. RNases

RNases, as a classical component of host defense, have been proposed as therapeutic candidates by catalyzing viral RNA. RNases, in addition to degrading viral RNA, play roles in IFN activation, viral replication inhibition, and apoptosis induction [21]. RNases have the potential to degrade viral genomes, but a major challenge is to limit host cell destruction. Numerous RNases exhibit antiviral properties against single-stranded RNA (ssRNA) viruses [22,23]. Here, we briefly described the RNase antiviral candidates promoted by viral RNA degradation. RNase A superfamily, EDN/RNase 2, cleaved the viral RNA against HIV-1 [24]. RNase L is induced by IFN to cleave cytoplasmic RNA through the OAS/RNase L signal pathway. RNase L is activated by 2′-5′ oligoadenylates (2–5A). OAS is activated by double-stranded RNA (dsRNA) binding to synthesize 2–5A. RNase L catalyzed viral RNAs of IAV [25] and WNV [26,27] in an in vitro experiment. Viral RNAs of HCV and DENV were digested by Regnase/MCPIP1 [28,29]. Recently, the antiviral RNases, such as OSA/RNase L have been well-characterized and exhibit multiple antiviral mechanisms. However, they present several challenges. There is incomplete cell protection against viruses via RNases, for instance, viruses escaping from RNases L have indicated the following in some cases: a neurotropic Theiler’s picornavirus produces a helper protein that inhibits RNases L [30], or DENV and IAV mRNAs escape RNase L-mediated decay, resulting in the production of viral protein [31]. RNases must be retained at the appropriate concentration to exhibit antiviral activity without exaggerated RNases cytotoxicity [32].

### 2.2. RNAi

RNAi is a cellular gene-silencing pathway that degrades the sequence-specific mRNA [33]. Since its discovery in 1998, RNAi has rapidly devolved as a therapeutic drug application with small interfering RNAs (siRNAs) being the most commonly used drugs. siRNAs have been widely studied against different viruses that degrade all types of viral genomes ds or ss DNA/RNA such as HIV [34,35], influenza virus [36,37], SARS-CoV [38,39], HBV [40,41], HPV [42], and WNV [43]. The FDA approved the first siRNA-based drug in 2018, followed by three more commercial drugs until now [44]. This was a significant step in siRNA research toward practicality. This indicated the potential of siRNAs as antiviral drugs in the future when some siRNA-based antiviral drugs entered the clinical phases, for instance, NucB1000 in phase I targets four HBV genes [45], ALN-RSV01 in phase II targets the nucleocapsid gene of RSV [46], pHIV7-shI-TAR-CCR5RZ in phase I targets multiple genes of HIV [33], TKM-Ebola in phase I targets multiple transcripts of Ebola virus [47]. The mechanism of siRNAs is as follows: after being taken up by the cell, numerous processes are performed by entering endosomes and following the endosomal escape. Then, siRNAs are released into the cytoplasm and taken up by the multiprotein RNA-induced silencing complex (RISC), and siRNAs guide the RISC complex to identify and cleave the targetable regions [44]. One of the barriers to its application is the delivery of siRNA to the target cells and cell penetration. In addition, siRNA technology has faced and overcome several challenges, such as poor stability, immunostimulation, off-target effects, viral escape due to mutations, and durable viral silencing to achieve sustainable antiviral therapy [33,44].

### 2.3. ISG20

IFN or dsRNA induces ISG20 expression and its presence in the cytoplasm and nucleus [48,49,50]. ISG20 is a 3′-5′ exonuclease that belongs to the DEDDh subgroup of the DEDD exonuclease superfamily [51]. ISG20 cleaved both ssRNA and DNA but showed a preference for RNA substrates over DNA by Met14 and Arg53 to accommodate hydrogen bonds with the 2′-OH group of UMP ribose [52,53]. Therefore, IGS20 exhibited greater antiviral activity to multiple RNA viruses than to DNA viruses [54] but failed to prevent SARS-CoV and DNA viruses, such as adenoviruses. Viral RNA degradation and direct or indirect inhibition of viral translation are the two proposed antiviral mechanisms of ISG20 (recently reviewed in [55]). The lack of viral inhibition by catalytically inactive ISG20 form-D94G supports the hypothesis that ISG20 exonuclease activity determines viral genome degradation. However, the specific action of viral genome degradation without damaging the host cells is yet to be investigated [52,56]. ISG20 has no apparent sequence specificity for viral genome degradation. However, in HBV infection, its overexpression does not cause cellular RNA degradation by the cellular cofactor YTHDF2. YTHDF2 is a cytoplasmic YT521-B homology domain-containing protein, the main group of m^6^A readers in the m^6^A pathway. The recruitment of ISG20 for RNA degradation through the binding of YTHDF2 to m^6^A modified the HBV pregenomic RNA. This study demonstrated that HBV RNA was selectively degraded by IGS20 [57,58,59,60]. 

Three factors regulate the selective binding of ISG20 to viral RNA of HBV: the appropriate specific binding site (the sequence GGACA of HBV ε loop), m^6^A modification, and the host factor, YTHDF2. Although this mechanism is specific to HBV, a similar scheme may be applied to other viral RNAs [57]. Most recent studies have shown the antiviral effect of ISG20 via the ectopic expression or generation of transgenic cell lines; therefore, it is necessary to focus on the mechanism underlying the intracellular delivery of ISG20 as an antiviral agent (Table 2).

The finding that ISG20 antiviral activity was not mediated by RNA degradation but rather by translation inhibition demonstrated the exonuclease-independent antiviral activities of ISG20. The overexpression of ISG20 correlated with the expression of IFIT1 preventing viral RNA translation with non-2′O-methylated 5′ caps [61]. The viral translation inhibition is mediated by ISG20 discrimination of self-nucleic acid from nonself [60]. Furthermore, ISG20 exhibited HBV replication inhibition via exonuclease-independent activities [56]. Obviously, ISG20 acquired multiple antiviral mechanisms, which prompted the development of a potential antiviral drug that is not limited to viral genome degradation.

**Table 2:** ISG20 exhibited an antiviral effect against viruses via viral genome degradation.

ISG20 Derivation	Virus	Genome	Replication	Genus	Family	Ref
Ectopic expression	HBV	Double-stranded, relaxed circular DNA (rcDNA)	Nucleus	*Orthohepadnavirus*	Hepadnaviridae	[56,57,58]
Ectopic expression	YFV	(+)ssRNA	Cytoplasm	*Flavivirus*	Flaviviridae	[62]
Transgenic cell line	BVDV	(+)ssRNA	Cytoplasm	*Pestivirus*	Flaviviridae	
Transgenic cell line	HAV	(+)ssRNA	Cytoplasm	*Hepatovirus*	Picornaviriade	
Transgenic cell line	VSV	(−)ssRNA	Cytoplasm	*Vesiculovirus*	Rhabdoviridae	[63]
Transgenic cell line	Influenza	(−)ssRNA	Nucleus	*Alphainfluenzavirus*	Orthomyxoviridae	
Transgenic cell line	EMCV	(+)ssRNA	Cytoplasm	*Cardiovirus*	Picornaviriade	
Transgenic cell line	WNV	(+)ssRNA	Cytoplasm	*Flavivirus*	Flaviviridae	[64]
Transgenic cell line	DENV	(+)ssRNA	Cytoplasm	*Flavivirus*	Flaviviridae	
Transgenic cell line	HCV	(+)ssRNA	Cytoplasm	*Hepacivirus*	Flaviviridae	[65]

### 2.4. CRISPR/Cas System

The clustered regularly interspaced short palindromic repeats (CRISPR)-Cas (CRISPR-associated proteins), known as the immune system of bacteria and archaea, has been a powerful tool for editing genes. Nevertheless, it has been developed as a CRISPR/Cas-based antiviral therapy, making it one of the potential candidates for viral treatment. In this section, we only focused on the feasibility of directly targeting the virus of CRISPR/Cas without modifying the host genome. CRISPR/Cas can be categorized into two main classes, with six types and several subtypes based on the effector Cas domain content [66,67]. Of these, class II Cas endonucleases have recently been a popular topic for antiviral studies, with the ability of dsDNA digestion of effectors, such as Cas9, Cas12, and ssRNA cutting of Cas13 [68]. The CRISPR/Cas system works on the principle that a single-guide RNA (sgRNA), after transcribing into small antisense CRISPR RNA (crRNA), guides the crRNA-Cas ribonucleoprotein complex, which eventually recognizes the specific sequences and destroys the viral targets (including intracellular viral genome and viral mRNAs) [67,68].

The CRISPR/Cas9 method is a promising tool for chronic diseases, as demonstrated by the studies shown in Table 3, which show decreased viral genome [69,70], declined latency reactivation [71,72,73], reduced GFP expression [71,72,73,74], and viral protein [72,74]. Within the six domains (REC I, REC II, Bridge Helix, PAM-interacting, HNH, and RuvC) of Cas9, the PAM-interacting domain binds to the sequence that matches its PAM after scanning the target DNA, and then the guide RNA (gRNA) complex melts the bases right upstream of PAM and complement them with the target on the gRNA. If this association occurs appropriately, RuvC and HNH-nuclease domains will cleave the target DNA after the third nucleotide upstream of PAM [66,75]. Similar to Cas9, Cas12 attacks dsDNA but has fewer off-target effects than Cas9. However, both require PAM with G-rich PAM for Cas9 and T-rich PAM for Cas12. The mechanism of Cas12 has some differences compared with that of Cas9 because Cas9 digests a target that is right next to PAM with the blunt-ended dsDNA breaks, but Cas12 exerts its effects downstream at some distance from PAM with sticky ends [76]. Although Cas9 can inhibit both DNA and RNA viruses by introducing DNA intermediates into the host cell, most of the viruses that infect humans are RNA viruses with more than 180 of 200 RNA viruses infecting humans [77,78,79]. Therefore, RNA-targeting CRISPR/Cas13 has been studied to address this issue. The Cas13 family contains at least four subtypes: Cas13a, b, c, and d. Unlike Cas9 and Cas12, Cas13 has a dual conserved higher eukaryote and prokaryote nucleotide-binding domain (HEPN) instead of the DNase domain (RuvC and HNH), as well as recognizes target RNA based on the PFS domain instead of the PAM domain [80]. Subsequently, target RNA is identified via PFS, and bound by gRNA, and Cas13 then cleaves the target RNA [80,81]. The first approach of Cas13 against SARS-CoV-2 in human cells (PACMAN-prophylactic antiviral CRISPR in human cells) used pan-coronavirus crRNAs; Abbott et al. discovered that six crRNAs could target 91% of the sequenced coronaviruses, leading to several other studies using Cas13-targeted SARS-CoV-2 and influenza virus, resulting in positive outcomes in vitro and in vivo [68,82,83]. Additionally, targeting positive-sense viruses results in the degradation of viral gene expression by Cas13 and the preparation of RNA for assembling new particles as well as viral RNA genome serving as templates for replication [68,82].

Apart from the apparent advantages, the main challenges include accurate delivery, high specificity, lower immunogenicity, and long-lasting effect on inoculated in vivo models. To date, there is consensus over whether Cas13 exhibits significant off-target effects. In most HIV research, no off-target effect has been reported, and the collateral damage is undetectable in many applications using Cas13s as the RNA targeting system in mammalian cells, animal models, and plants [75,84,85,86,87,88,89,90]. Nonetheless, the extent of the off-target effects indicated the differences based on the cell type and target RNA observed. For instance, Yuxi Ai et al. showed that the extension of the off-target effects of CRISPR/Cas13s limited their utility in eukaryotic cells [91]. Therefore, this notion remains a challenge limiting their clinical trial. Notably, Cas13 research design and interpretation should be carefully performed to achieve potential results [91].

To use the CRISPR/Cas system, sgRNAs must be designed and constructed based on highly conserved regions of the viral genome to avoid escape mutants and reduce the possibility of off-target effects. This is followed by cloning these sgRNAs into an expression plasmid or viral vector, and then expressed using lipofectamine transfection or lentiviral/adenoviral transduction [68].

**Table 3:** Viral genome-specific cleavage by CRISPR with different Cas effector.

Cas	Experiment	Target Gene	Virus	Genome	Replication	Genus	Family	Ref.
Cas9	WSL-gRp30 cell	p30 gene (CP204L)	ASFV (BA71V)	dsDNA	Cytoplasm	*Asfivirus*	Asfarviridae	[92]
	Vero, ICP0-complementing L7 cell line 27, TC620	ICP0, ICP4, and ICP27 genes	HSV-1	dsDNA	Nucleus	*Simplexvirus*	Herpesviridae	[93]
	Vero, 239T, and BALB/c mice	UL7 genes						[94]
	Vero cell	UL15, UL27, UL29, UL30, UL36, UL37, UL42, UL5, UL52, UL8, UL54, UL9, US3, and US8						[95]
	Vero cell	UL8, UL29, and UL52						[96]
	297T, HaCaT, HaCaT IFNAR2-knockout, THP-1, primary mouse corneal stromal cell, and C57BL/6J mice	UL8 and UL29 genes						[97]
	HEK293T, HeLa, and Jurkat c5 and c19 cells	TLR	HIV-1	(−)ssRNA	Reverse transcription in cytoplasm Replication in nucleus	*Lentivirus*	Retrovirus	[82]
	CHME5 cell, HeLa-derived TZM-bI cells, promonocytic U-937 cell subclone U1	TLR-U3						[83]
	Transgenic mice	5′-LTR and Gag gene						[98]
	Human T-lymphoid cell, Jurkat 2D10, PBMCs	LTR-U3						[99]
	NRG mice	LTR						[71]
	HEK293T, Jurkat C11, and TZM-bl cells	LTR7, LTR8, and structural region (env5, vif2, rev3, gag8, pol6, and pol7)						[100]
	HEK293T	LTR, gag, and pol						[84]
	Tg26 transgenic mice, BLT mice, NCr nude mice	LTR, gag, and pol						[101]
	HEK293FT, primary human monocytes	LTR, gag, env, ref, tat						[102]
Cas12a	HEK293T cell	LTR, gag, env, pol, tat, rev, nef, vpr						[70]
Cas13a	HEK293T cell, HEK293 cell	LTR, gag, tat, and rev						[103]
Cas9	HepG2.2.15 cell	HBV DNA sequences	HBV	Double-stranded, relaxed circular DNA (rcDNA)	Nucleus	*Orthohepadnavirus*	Hepadnaviridae	[72]
	HepG2 cell, and Balb/c mice	Conserved regions of HBV						[74]
	Huh7 cell, HepG2.2.15 cell, and Balb/c mice	20 nucleotide HBV DNA sequences						[104]
	HepG2 cell	Conserved regions of HBV						[105]
	Huh7 cell, and C57BL/6 mice	Conserved regions of HBV						[106]
	PK-15 cell	UL30	PRV	dsDNA	Nucleus	*Varicellovirus*	Herpesviridae	[73]
	Vero cell	Essential and nonessential genes						[107]
	Vero cell	EBNA1, OriP	EBV	DNA	Nucleus	*Lymphocryptovirus*	Herpesviridae	[95]
	Vero cell	UL54, UL44, UL57, UL70, UL105, UL86, and UL84	HCMV	DNA	Nucleus	*Cytomegalovirus*	Herpesviridae	[95]
Cas9	Hela cell, Caski, HEK293T, Jurkat, Hela-FLAG16E7MYC cell, and Rag1 mice	E6 and E7	HPV	dsDNA	Nucleus	*Alphapapillomavirus*	Papovaviridae	[108]
	SiHa, C33-A, and BALB/c nude mice	E6 and E7						[109]
	SiHa cell, and nude mice	E7						[110]
	Hela, HCS-2, SKG-I, 293, and BALB/c nude mice	E6						[111]
	Hela, 293T, and SiHa cell	E6 and E7	HPV	dsDNA	Nucleus	*Alphapapillomavirus*	Papovaviridae	[112]
Cas12a	BmN-SWU1 cell, and transgenic silkworm	ei-1 gene	BmNPV	dsDNA	Nucleus	*Alphabaculovirus*	Baculovirudae	[69]
Cas13a	Mice	PB1 and PB2 genes of influenza	IAV-H1N1 (A/WSN/33)	(−)ssRNA	Nucleus	*Alphainfluenzavirus*	Orthomyxoviridae	[89]
	MDCK cell	Conserved regions of H1N1	IAV-H1N1 (A/Puerto Rico/8/1934)	(−)ssRNA	Nucleus	*Alphainfluenzavirus*	Orthomyxoviridae	[81]
	A549 cell	Conserved regions of H1N1	IAV-H1N1	(−)ssRNA	Nucleus	*Alphainfluenzavirus*	Orthomyxoviridae	[77]
	Hamsters	Replicase and nucleocapsid genes of SARS	SARS-CoV2	(+)ssRNA	Cytoplasm	*Betacoronavirus*	Coronaviridae	[89]
	HepG2 cell, and AT2 cell	S gene	SARS-CoV2					[113]
	A549 cell, and HEK293T cell	RdRp (ORF1ab) and N gene	SARS-CoV2	(+)ssRNA	Cytoplasm	*Betacoronavirus*	Coronaviridae	[77]
	-	Replicase and transcriptase (ORFab) and S gene	SARS-CoV2	(+)ssRNA	Cytoplasm	*Betacoronavirus*	Coronaviridae	[114]
	HEK293FT cell	Conserved regions of LCMV	Wild type-LCMV Armstrong	(−)ssRNA	Cytoplasm	*Mammarenavirus*	Arenaviridae	[81]
	HEK293FT cell	Conserved regions of VSV	VSV	(−)ssRNA	Cytoplasm	*Vesiculovirus*	Rhabdoviridae	[81]
	HEK293T, HEK293FT, and MARC-145 cell	ORF5 and ORF7	PRRSV	(+)ssRNA	Cytoplasm	*Porartevirus*	Arteriviridae	[115]

### 2.5. 3D8 Single-Chain Variable Fragment (3D8 scFv)

A monoclonal antibody (mAb) named 3D8 was discovered and isolated as an anti-DNA Abs from the spleen cells of MRL-*lpr/lpr* mice, an autoimmune-prone mouse model that resembles human systemic lupus erythematosus. Sequentially, a recombinant 3D8 single-chain variable fragment (3D8 scFv), 27kD, was generated in the formation of the heavy chain variable single domain (VH) connecting the light chain variable single domain (VL) via a flexible (Glycine4-Serine1)3 linker [116]. The 3D8 scFv protein can bind and hydrolyze nonspecific on both DNA (dsDNA, ssDNA) and RNA (dsRNA, ssRNA) in the presence of Mg^2+^ [117,118]. Although methylated and histone-bound DNA were not cleaved by 3D8 scFv, viral RNA hydrolyzed in vRNP form was recently reported [119,120]. The 3D8 scFv protein can be obtained from different systems, and the protein retains functional activities such as *E. coli* bacterial expression system [116,117], *Lactobacillus paracasei* [121], or in vegetatively reproductive kalanchoe pinnata via planta transformation [122]. However, human embryonic kidney 293f (HEK293f)-derived 3D8-His scFvs lost all DNA-hydrolyzing activity but retained its DNA-binding activity [123]. Unlike other cell-penetrating anti-DNA, Abs reported so far eventually traffic toward the nucleus. The 3D8 scFv can penetrate cells via caveolae/lipid raft endocytosis, which is mediated by heparan sulfate proteoglycans (HSPGs) and chondroitin sulfate proteoglycans (CSPGs) that function as endocytic receptors on the cell surface [124] and accumulate and remain up to 48 h in the cytosol without further translocation into endosomes, lysosomes, endoplasmic reticulum, Golgi, or nucleus [125]. Organ penetration of 3D8 scFv in vivo was revealed by its localization in the intestinal villi and lamina propria [121], epithelial cells, medium diameter bronchi and alveoli [126], and muscle, liver, lung, and brain tissues [118]. Furthermore, 3D8 scFv was maintained in the lung for 12 h by IN injection; in the liver, kidney, plasma, and lung for up to 6 h by IV or IP injection. An increase in the injection frequency extended 3D8 scFv retention time in the organ. In all transgenic chicken, mice, plants, or stable cell lines harboring the 3D8 scFv gene, the protein concentration was expressed as too low to be detectable, which is sufficient for conferring antiviral effects without incurring damage to host DNA/RNA. A sufficient 3D8 scFv dose is critical to target only viral DNA and RNA but not their host genetic material. Nucleic acid hydrolyzing 3D8 scFv treatment exerted some cytotoxic effects on cells because of its ability to hydrolyze cellular RNAs at high concentrations [125]. A treatment with 5 μM of 3D8 scFv caused no cytotoxicity for 48 h in cells [127]. There were no dead mice found for up to 5 d at 20, 50 μg [118,126], or up to 300 μg of 3D8 scFv injection [120]. Nucleic acid-hydrolyzing catalytic and cell penetration or organ distribution activities of 3D8 scFv has prompted its investigation in multiple antiviral applications. Interestingly, 3D8 scFv has shown antiviral activity against a broad spectrum of DNA and RNA viruses in both in vitro and in vivo experiments (Table 4). As a result, 3D8 scFv can be applied as a feed additive. Assessment for the use of purified 3D8 scFv as a feed additive according to the concept of substantial equivalence was confirmed, in which oral injection of *E. coli* 3D8 scFv was excreted from mice within the normal digestive transit time of the animals without colonization to the gastrointestinal tract [128]. Moreover, the administration of *Lactobacillus salivarius* expressing 3D8 scFv or *Lactobacillus reuteri* harboring 3D8 scFv as a feed additive enhances growth performance, immune homeostasis, and gut microbiota of chickens [129,130]. A metagenomic analysis revealed that probiotic *Lactobacillus paracasei* expressing 3D8 scFv enhances the probiotic activities in mice without any observable side effects [131]. 3D8scFv that originated from mouse (m3D8 scFv) were humanized (h3D8 scFv) or chickenized (ck3D8 scFv). Both h3D8 scFv and ck3D8 scFv retained the biochemical properties of m3D8 scFv as well as the structure. Importantly, ck3D8 scFv expressed lower immunogenicity than m3D8 scFv in chickens [132,133].

## 3. Discussion

**Figure 1:**
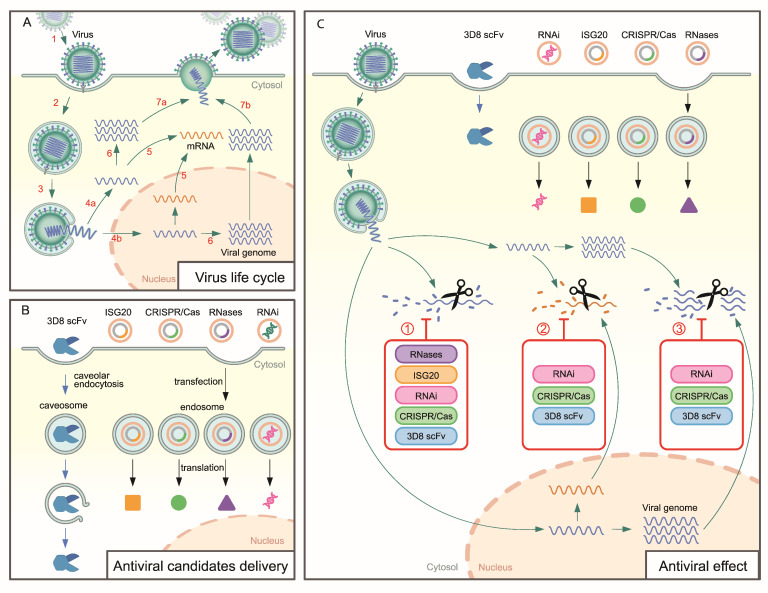
Proposed mechanism of viral genome degradation-specific antiviral candidates. (**A**) Typical viral life cycle of enveloped viruses; virus infection to host cells includes several main steps, 1. Binding; 2. Fusion; 3. Release of viral genome in the cytoplasm; 4a. Replication in the cytoplasmic or 4b in the nucleus (viral genome needs to be transported to the nucleus), 5. Transcription and presence of mRNA in the cytoplasm for translation; 6. Viral genome replication; 7a, 7b. Assembly and release of new virions. (**B**) 3D8 scFv with cell penetration ability via caveolar endocytosis and localization in the cytoplasm, intracellular localization of other candidates (CRISPR/Cas, ISG20, RNases, and RNAi) are proposed with a delivery system and expressed as protein inside the cells. (**C**) The presence of viral genomes cleavage candidates (3D8 scFv, CRISPR/Cas, ISG20, RNases, RNAi) inhibits viral replication. Upon infection, viral genomes distributed in the cytoplasm were digested by RNase, ISG20, CRISPR/Cas, 3D8 scFv, and RNAi. 3D8 scFv, RNAi, and CRISPR/Cas were supposed to degrade viral mRNA in the cytoplasm for translation. Finally, the replicated viral genomes can be catalyzed using 3D8 scFv, RNAi, and CRISPR/Cas.

Since the approval of idoxuridin, the first approved antiviral drug for the treatment of hepatitis B in 1963, many approved antiviral drugs have been commercially available for use. These antiviral drugs have a mode of action wherein they target the major stage of the viral life cycle to interfere with its replication process, as summarized in Figure 1A [15]. Based on the understanding of virology, antiviral drugs have been developed and are concentrated on two different approaches: targeting the host cell factors or the viral life cycle at protein levels [11].

In this review, we take the opportunity to propose a novel approach for antiviral drug degradation of the viral genome, which can be applied against various broad-spectrum viruses (Figure 1). From a perspective used as an antiviral approach, it is necessary to ensure that these candidates are correctly delivered to the target. Antiviral activity of these candidates in the in vitro and in vivo experiments was obtained via the ectopic expression by transient transfection or generation of transgenic cell lines, and animals. Undoubtedly, the use of suitable delivery vehicle tools by transfection, viral genome catalytic candidates, RNases, RNAi, and ISG20 or CRISPR/Cas, could expand the antiviral activity. Furthermore, 3D8 scFv was shown to penetrate cells, and it was localized in the cell cytoplasm via caveolae/lipid raft-mediated endocytosis [124,125] (Figure 1B). Each candidate can target different viral genomes stages, as indicated in (Figure 1C). As described, the presence of these candidates in the cytoplasm can digest the viral genome at the (1) releasing step, (2) viral mRNA transcription, or (3) replicated viral genomes for viral assembly.

The discovery of broad-spectrum antiviral agents, or “one drug, multiple viruses,” have been developed to protect us from various unknown and emerging viruses. However, a major challenge in developing antiviral drugs is a viral mutation, such as antigenic drift and antigenic shift in influenza virus, which causes the emergence of novel and drug-resistant strains of viruses. Other problems include the time and cost required to develop a new drug. Therefore, we aimed to introduce potential universal antiviral therapeutic targeting to digest viral genomes, a direct approach to inhibit viral replication. Notably, each candidate presents specific disadvantages that prompted further studies. For example, the unspecific cleavage site of RNases, ISG20, and 3D8 scFv encountered difficulty in discriminating viral genome and host cell nucleic acid, leading to the death of infected cells. Although many studies reported that a certain amount of 3D8 scFv demonstrated antiviral activity to various viruses and was not harmful to host cells, the selectively targeted viral genome requires further modification. Additionally, RNases and ISG20 exhibited antiviral activity not only by degrading the viral genome but also by relating to other pathways. RNases regulate the host immune, the formation of stress granules (SGs), inducing autophagy, and triggering apoptosis [21,22]. ISG20 inhibited HBV replication by exonuclease-dependent and -independent activities [56]. There are several challenges in RNAi application, such as, delivery to target cells, stability, and virus evolution [33,44]. In the case of the CRISPR/Cas system recognizing specific in the viral genome, CRISPR/Cas9, Cas13 decreases the cleavage activity in the existence of two or more mismatches between target RNA and crRNA [142]. There have been several challenges, such as delivery route, standard viral delivery vectors, sgRNA design, off-target effects, safety, and immunogenicity, which need to be overcome to achieve the further success of the CRISPR/Cas: approach to the supply chain, clinical development, and commercialization, with the researchers’ efforts, CRISPR/Cas has been refined over time. However, it could offer a powerful antiviral treatment against emerging, re-emerging viruses in the near future.

In conclusion, we introduced a novel approach exhibiting direct and universal antiviral therapy by cleavage of the viral genomes, regardless of the type of viruses. RNases, ISG20, a catalytic nucleic acid hydrolyzing 3D8 scFv, RNAi, and CRISPR/Cas systems that target viral genome degradation have been shown to exhibit antiviral activity. We reviewed all aspects of the candidates, and proposed insight mechanisms for each one to be considered as antiviral therapy.

## Figures and Tables

**Table 1:** The global epidemic and pandemic of viral diseases throughout history.

No.	Year ^1^	Disease	Virus	Death ^2^
1	1520	Smallpox	Variola virus	500 million
2	~1800	Yellow fever	Yellow fever virus	>210,000
3	1918	Spanish flu	Influenza A virus (H1N1)	50 million
4	1957	Asian flu	Influenza A virus (H2N2)	2 million
5	1968	Hong Kong flu	Influenza A virus (H3N2)	1 million
6	1976	Ebola	Ebola virus	~15,300
7	1981	HIV/AIDS	HIV	~37 million
8	1990	Dengue fever	Dengue virus	>680,000
9	2002	SARS	SARS-CoV	774
10	2009	Swine flu	Influenza A virus (H1N1)	284,000
11	2012	MERS	MERS-CoV	891
12	2014	Chikungunya	Chikungunya virus	rare
13	2015	Zika	Zika virus	~1000
14	2020	COVID-19	SARS-CoV-2	~6.2 million

^1^ Refer to the year of the first outbreak case, ^2^ refer to the estimated number of deaths until recently.

**Table 4:** 3D8 scFv hydrolyzes viral genomes exhibiting antiviral activity against broad-spectrum viruses.

Derivation	Experiment	Virus	Genome	Replication	Genus	Family	Ref.
Protein expressed in *E. coli*	PK-15 cells	CSFV	(+)ssRNA	Cytoplasm	*Pestivirus*	Flaviviridae	[127]
Transgenic cell line							
Protein expressed in *E. coli*	Hela cells	VSV	(−)ssRNA	Cytoplasm	*Vesiculovirus*	Rhabdoviridae	[134]
Transgenic plants	*N. tabacum*	PMMoV	(+)ssRNA	Cytoplasm	*Tobamovirus*	Virgaviridae	[135]
		TMGMV					
		ToMV					
		TMV					
		CMV			*Cucumovirus*	Bromoviridae	[135]
Transgenic plants	Chrysanthemums	CSVd	(−)ssRNA	Nucleus	*Pospiviroid*	Pospiviroidae	[136]
Protein expressed in *L. paracase*	RAW264.7 cells	MNV1	(+)ssRNA	Cytoplasm	*Norovirus*	Calciviridae	[121]
Transgenic bacteria *L. paracase*	Mice						
Protein expressed in *E. coli*	MDCK cell	H1N1/NWS33	(−)ssRNA	Nucleus	*Influenzavirus A*	Orthomyxoviridae	[120]
		H9N2					
		H1N1/PR8					
		H3N2					
	MDCK cell/Mice	H1N1/09pdm					
Transgenic animal	Chickens	H9N2	(−)ssRNA	Nucleus	*Influenzavirus A*	Orthomyxoviridae	[137]
Transgenic animal		Infectious bronchitis virus	(+)ssRNA	Cytoplasm	*Gammacoronavirus*	Coronaviridae	[138]
Transgenic animal		Newcastle disease	(−)ssRNA	Cytoplasm	*Avulavirus*	Paramyxoviridae	[139]
Protein expressed in *E. coli*	Vero E6	SARS-CoV-2	(+)ssRNA	Cytoplasm	*Betacoronavirus*	Coronaviridae	[140]
		hCo-OC43			*Betacoronavirus*	Coronaviridae	
		PEDV			*Alphacoronavirus*	Coronaviridae	
Transgenic cell line	Hela	HSV1	dsDNA	Nucleus	*Simplexvirus*	Herpesviridae	[119]
Transgenic cell line		PRV	dsDNA	Nucleus	*Varicellovirus*	Herpesviridae	[119]
Transgenic animal	Mice		dsDNA	Nucleus	*Varicellovirus*	Herpesviridae	[118]
Transgenic plants	*N. tabacum*	BCTV	ssDNA	Nucleus	*Curtovirus*	Geminiviridae	[141]
Transgenic plants	*N. tabacum*	BSCTV					
